# One Step at a Time: Representational Overlap Between Active Voice, Be-passive, and Get-passive Forms in English

**DOI:** 10.5334/joc.36

**Published:** 2018-06-26

**Authors:** Dominic Thompson, Fernanda Ferreira, Christoph Scheepers

**Affiliations:** 1 School of English, University of Nottingham, Nottingham, UK; 2 Department of Psychology, University of California, Davis, CA, US; 3 Institute of Neuroscience and Psychology, University of Glasgow, Glasgow, UK

**Keywords:** sentence processing, language production, language comprehension, mental representation, passives, syntax

## Abstract

The active voice and passive voice are complementary sentence forms that are available when describing a transitive event. In English, the latter has two variants: be-passive and get-passive. Numerous attempts have been made in the literature to represent the syntactic and semantic differences between these forms, while maintaining their shared features, yet theoretical accounts still differ. At the same time, empirical studies into structural choice have frequently investigated the use of passive voice versus active voice, while the distinction between get- versus be-passive has not received much attention.

Here we investigate the degree of similarity between the three transitive variants (be-passive, get-passive, active voice), providing experimental evidence of their mental representations in relation to each other. We describe three experiments in which participants gave acceptability or naturalness ratings for sentences formed with either be-passive or get-passive, and containing one of several adjunct types. Participants were also free to provide an alternative way to phrase each, enabling us to consider whether there are differences in accessing alternatives.

We observed overwhelming preferences for changing get-passives into be-passives, and for changing be-passives into active voice, but none for changing get-passives directly into active voice (despite active voice being the most preferred variant). This preference for changing get-passive into be-passive was observed even when a change into active voice was further facilitated by the availability of a ‘ready-made’ agent.

These patterns of change are consistent with partial representational overlap along two dimensions described by Thompson et al. (2013): Patient Prominence and Patient Importance. Our findings also contribute to discussions of passive structure by revealing the relative closeness of the mental representations of these forms.

## Introduction

In many languages, the passive voice is a linguistic construction providing speakers with an alternative to the more frequent active form. While both can be used to describe transitive events, active voice generally forces the *Agent* (the person who does the action) to assume the role of the grammatical subject of the sentence. The primary function of the passive voice is to allow the *Patient* or ‘undergoer’ of an action to appear as the grammatical subject instead, which, in English (as well as other ‘positional’ languages without strong case morphology), facilitates appearance in sentence-initial position (Keenan & Dryer, 2006).^1^ A speaker may place the patient in subject position to maintain it as the topic of a discourse, or alternatively to provide it with added attention or mark it as important. For example, speakers are more likely to assign importance to an animate entity than to an inanimate one, therefore preferring to give the animate entity a prominent syntactic role (for recent experimental evidence, see Gennari, Mirković, & MacDonald, 2012). Here we consider agents and patients with no inherent difference in importance, using only animate, human protagonists.

In English, the passive voice features two major variants: be-passive (as in *Mary was bitten [by a dog]*.) and get-passive (*Mary got bitten [by a dog]*.). Corpus analyses show that the get-passive is a more recent addition to English than the be-passive (e.g. Hundt & Mair, 1999; Hundt, 2001; Mair & Leech, 2006), and is also increasing in use. Indeed, Hundt & Mair (1999) observed that over the 30-year period between 1961 and 1991, there was a significant proportional decline in the use of be-passives, while at the same time, get-passive uses (though relatively infrequent overall) nearly doubled in frequency. Interestingly, these longitudinal trends in get- versus be-passive use, as well as the underlying structural frequencies, were nearly the same between reference corpora of American and British English.

Though occasionally suggested to be equivalent in syntax (Chomsky, 1981) and in semantics (Weiner & Labov, 1983), there is general recognition that get- and be-passives behave differently and have their own preferred uses. The general syntactic structure of the be-passive is well-established, with the thematic patient raising up the structure in order to satisfy its case requirement: [Ellie_i_ was *t*_i_ hired *t*_i_].^2^

The get-passive can be conceptualised as following a similar ‘raising’ construction, where the thematic patient raises from object position into subject position (Haegeman, 1985; Fleisher, 2008), as in: [Ellie_i_ *t*_i_ got hired *t*_i_]. In this type of approach, be-passives and get-passives are similar in that they both serve to allow movement of the patient into subject position. Nonetheless, the two passive-types are still assumed be structurally distinct in order to account for their differing syntactic behaviour, as first highlighted by Haegeman (1985). An alternative type of approach proposes that passive *get* is a subject control verb: a verb that forces the covert (unpronounced) subject of an embedded clause to refer to the subject of the matrix (main) clause (Huang, 1999; Butler & Tsoulas, 2006), as in: [Ellie_i_ got PRO_i_ hired *t*_i_]. This type of ‘control’ approach to the get-passive means that, while the two passive-types share the semantic function of having the subject refer to the patient, they are structurally quite distinct.

The current overriding analysis is of a raising approach that involves variants or layers of ‘small v’ phrases, into which aspects such as ‘voice’ are coded (Wanner, 2009; Alexiadou, 2012; Alexiadou & Schäfer, 2013). However, discussions continue regarding how the two forms differ, and the precise function of each. To drive these discussions forward, more systematic experimental research is needed.

Here we investigate the degree of similarity between the three variants of transitive event descriptions (be-passive, get-passive, active voice), with the aim of providing experimental evidence of how they are mentally represented in relation to each other. This would inform linguistic and psycholinguistic theories of passivization as well as syntax and semantics more broadly. By allowing participants to provide an alternative way to phrase various sentences in addition to standard acceptability judgments, we can assess whether there are differences in accessing alternatives to each of the variants.

The passive itself has been extensively studied in the language comprehension and production literature. There have been many investigations into the effects of memory on passive production, as well as differences in interpretation and processing difficulty of active versus passive sentences (e.g. Forster & Olbrei, 1972; Gough, 1965; Mehler, 1963; Savin & Perchonock, 1965; Slobin, 1966). Passives have also been the focus of research on structural priming (e.g. Bock, Dell, Chang, & Onishi, 2007; Olson & Filby, 1972), the neurophysiology of comprehension and production (e.g. Segaert et al., 2012), non-canonical language use (e.g. Ferreira, 2003), and language acquisition (e.g. Whitehurst, Ironsmith, & Goldfein, 1974). However, the important distinction between be-passive and get-passive has largely been disregarded. The primary exceptions to this are in the developmental literature, in which it is young children’s understanding and acquisition of get-passive versus be-passive that are examined (see for example, Brooks & Tomasello, 1999; Fox & Grodzinsky, 1998; Marchman, Bates, Burkardt, & Good, 1991; Meints, 2003; Messenger et al., 2008).

In terms of get-passives, a few broad theoretical points recur in the linguistic and psycholinguistic literature. Firstly, the get-passive is often noted as placing greater focus on the event or its outcome than the be-passive (Alexiadou, 2005; Carter & McCarthy, 1999; Cheshire, 2005; Palmer, 1974; Sasaki, 1999). This is supported by corpus data revealing the get-passive’s tendency to appear disproportionately more frequently without an agentive by-phrase (Carter & McCarthy, 1999; Collins, 1996; Medina, 2009; Mindt, 2000; Rühlemann, 2007). One also finds that the patient of a get-passive is usually identified as being more ‘affected’^3^ than the patient of a be-passive (Cameron, 1996; Carter & McCarthy, 1999; Orfitelli, 2011; Sasaki, 1999). Relatedly, the patient often tends to be apportioned some level of blame in the get-passive that is absent in a corresponding be-passive (Arrese, 1997; Barber, 1975; Cameron, 1996; Downing, 1996; Givon & Yang, 1994; Hatcher, 1949; Lakoff, 1971; Lasnik & Fiengo, 1974; Vanrespaille, 1991; Sasaki, 1999). Finally, an important feature of the get-passive is its greater semantic range in comparison to the be-passive, allowing it to achieve the meanings and connotations above, and additional ones described in the literature (Alexiadou, 2005; Givon & Yang, 1994; Sussex, 1982; Thompson, 2013; Thompson & Scheepers, 2013). For a more comprehensive summary and analysis of the relevant literature, see Thompson, Ling, Myachykov, Ferreira, & Scheepers (2013) and Thompson & Scheepers (2013).

As has been discussed in detail elsewhere (Thompson et al., 2013; Thompson & Scheepers, 2013), there is strong agreement throughout the literature regarding the underlying syntactic structure of the be-passive, as well as its general semantics. However, there remain extensive variations and on-going discussion concerning the syntax and semantics of the get-passive. Approaches to get-passive semantics tend either to equate it with the be-passive, or search for one precise meaning or function specific to the get-passive (as discussed in Wanner, 2009).

Despite the generally conflicting suggestions in the literature of how get-passive semantics differ from be-passive semantics, many can be categorised as claiming either that *get* assigns some general importance to the event’s patient, or that it marks some level of patient agency. To explore this, Thompson et al. (2013) conducted a series of paraphrasing experiments in which participants retold short stories in their own words. The critical parts of the stories were always in active voice, but were manipulated to either place the event’s agent or patient into focus, either via an earlier mention in the story (a ‘given’ versus ‘new’ agent or patient), syntactic clefting (e.g., *It was her who the manager hired*), or question formulation (e.g., *What happened to [Patient]?*). These experiments aimed to investigate how the above factors influence the production of active voice and passive voice, and the selection between be-passive and get-passive. Briefly, their findings were that a topical agent (i.e., an agent that is already introduced by prior discourse) promotes the use of active voice, while a topical patient promotes the use of *be-passives specifically*, rather than passives in general. Furthermore, the use of get-passives (but not of be-passives) rises in response to a patient that is marked as important (e.g., via syntactic clefting).

Based on these findings, the authors proposed that the three transitive variants in English can be arranged along two dimensions. The first is *Patient Prominence*, referring to the ‘functional prominence’ of the patient, as indicated by its assignment to a prominent syntactic role such as the subject position in English. This is realised via the choice between active voice and passive voice. The second dimension is *Patient Importance*, referring to focus or importance of the patient in the event beyond discourse topicality. This is realised via the choice between be-passive and get-passive. Table 1 indicates how the interplay between these two dimensions uniquely describes active voice, be-passive, and get-passive. The fourth cell in the table remains somewhat unclear at present. It could be that there is no form in English that directly maps onto a scenario whereby an important Patient is not assigned a prominent (Subject-) role. One possibility suggested by the authors for this cell is that of a clefted active voice construction (e.g., *It was her who the manager hired*). Given the current uncertainty, and the more complex syntax of this construction, we do not consider it in the present work.

**Table 1:** Distribution of the three transitive description variants (active voice, be-passive, get-passive). These are shown along two dimensions (Patient Importance and Patient Prominence) (adapted from Thompson et al., 2013).

		Patient Prominence	

		+	–

**Patient Importance**	+	Get-passive	/
	–	Be-Passive	Active

According to this description, the be-passive and get-passive share one attribute: the placement of the event’s patient in the prominent subject position; however, it is only the get-passive that further marks the patient as important. Likewise, active voice and the be-passive share a non-important patient (i.e., the agent is more important in those forms); however, the be-passive has a functionally prominent patient, while active voice has a non-prominent patient (instead having a functionally prominent agent). Active voice and the get-passive do not share either of these attributes. Note that the above proposal is rather informal and does not make claims about more precise structural or semantic differences among the forms; instead, its aim is to highlight the most notable factors that distinguish these forms in mental representations.

## Objectives and Hypotheses

Our aim is to further understand how the three transitive variants overlap in mental representation. Whereas the above studies by Thompson et al. (2013) considered factors that drive the selection of one passive-type over the other (by encouraging paraphrases of original active voice sentences), here we look at paraphrases between be-passive and get-passive, and from each passive-type into active voice, with a view to revealing how these three variants are mentally represented in relation to each other. We will also consider the additional influence of various types of adjunct, the inclusion of an agent, the inclusion of the preposition ‘by’, and combinations of these. To do this, we will use rating tasks to establish relative preference for each passive-type by adjunct-type combination. This will be followed by an optional paraphrasing task, allowing participants to suggest an alternative way to say each sentence. The latter option to ‘change’ the sentences provides insight into underlying representational similarities between different variants of transitive description.

Assuming the two dimensions discussed above, we predict that changes between transitive variants will be easiest when only a single dimension is altered in the process; that is, when there is greater *representational similarity* between variants. Changes between get-passive and be-passive (alteration in Patient Importance only), and between be-passive and active voice (alteration in Patient Prominence only), should be easier (and hence more frequent) than changes between get-passive and active voice, which involves changes to both of these dimensions.

We expect be-passives to be rated as more acceptable or more ‘normal’ than get-passives, given their more frequent occurrence in corpora (e.g. Chafe, 1982; Mair & Leech, 2006). From this, we can also hypothesize that when asked for a ‘better way’ to say the sentences, participants should be more likely to change get-passives into the (more preferred) be-passive form than be-passives into the (less preferred) get-passive form. In contrast, when asked simply for a ‘different’ way to say the sentences, there may be less directionality; that is, be-passives may be changed ‘up’ to active voice as frequently as they may be changed ‘down’ to get-passives.

These patterns of change may be modulated by the presence or absence of an adjunct, and the type of adjunct that is present. Truncated passives are likely to be the preferred form, as they are again the most frequently occurring in corpora (Carter & McCarthy, 1999; Xiao, McEnery, & Qian, 2006). Moreover, the inclusion of an agentive by-phrase (such as *by the driver*) should make it easier to form the active voice. This is particularly evident when one considers that the change of a truncated passive into an active voice sentence requires the potentially more effortful use of a generic Agent such as “*they*” or “*someone*” (e.g., *Mary was attacked* → *Someone attacked Mary*). Therefore, the presence of a ‘ready-made’ agent should increase the frequency of changes into active voice in one of two ways: (a) if representational similarity has a *strong* influence, then the presence of an agent should boost the likelihood of changes into active voice specifically for be-passives, and to a lesser extent for get-passives (resulting in an interaction between Truncation and Passive-type); or (b) if representational similarity has a *weak* influence, the presence of an agent should boost changes into active voice equally for get-passives and be-passives (resulting in a Truncation main effect, but no interaction with Passive-type).

It has previously been demonstrated that a sentence with a locative by-phrase (such as *…by the riverside*) can prime the use of a passive that contains an agentive by-phrase (Bock & Loebell, 1990). Since an agent is most commonly introduced in the passive via a by-phrase, the presence of *by* alone may cue thoughts of an agent (though none is provided), hence a passive containing a *non-agentive* by-phrase may facilitate changes into active voice (though not to the degree observed when a ready-made agent is also included). On the other hand, Liversedge, Pickering, Branigan, & van Gompel (1998) showed in two eye-tracking studies that it is more difficult to process a locative rather than an agentive by-phrase, apparently because readers initially interpret all by-phrases as referring to agents in passive constructions (i.e., based on the verb morphology). Given these findings, the added difficulty of a non-agentive by-phrase may act as a distraction, resulting in changes that focus on the by-phrase rather than the transitive variants.

Experiment 1 examines differences between be-passive and get-passive, as well as the presence or absence of an agentive by-phrase. When there is no agentive by-phrase, the passive sentence is left truncated with no other adjunct in its place, as in *John got hired*. This establishes general preferences for each of the passive forms, by-phrase inclusion, and any interaction between the two, as well as testing the representational overlap between these forms.

Experiment 2 again considers both be-passives and get-passives, this time combined with one of three adjunct types: an agentive by-phrase (as in Experiment 1), a non-agentive by-phrase, and a non-by adjunct. This allows us to compare the effects of the presence and absence of an agent, as well as the presence and absence of the preposition ‘by’.

Experiment 3 follows the structure of Experiment 1, but in place of asking about acceptability, participants were asked how natural or normal the sentences seemed to them. Also, rather than prompting a ‘better way’ to say each sentence, participants are asked simply if they would say the sentence in ‘a different way’.

## Experiment 1

Experiment 1 orthogonally manipulated passive-type and by-phrase inclusion in a 2 × 2 design. This allowed us to consider the passive in its most basic and most commonly occurring forms, as well as to compare the effects of presence versus absence of an agentive by-phrase. Ratings provided a solid picture of perceived acceptability for each of the four forms, while free-responses explored the representational similarities between the three transitive variants.

### Participants

Eighty native-English speakers (age 17–63, mean age 28; 50% females) were tested in an online procedure. Participation in the study was voluntary and held no monetary payment. Participants were either undergraduate students at the University of Glasgow, or recruited through the University’s subject database. The studies in the present paper were approved by the ethics committee of the College of Science and Engineering at the University of Glasgow. Participants gave informed consent prior to participation, and were free to withdraw at any time without penalty.

### Stimuli

Sixteen material sets (Table 2) were created, each consisting of a passive voice sentence in four versions. The different versions were formed using either *be* or *get*, and either included an agentive by-phrase (full passive) or not (truncated). Truncated sentences had no other form of adjunct in place of the agentive by-phrase. The first conditional manipulation is referred to as *Passive-Type* (2 levels: *be* vs. *get*) and the second as *Adjunct Type* (2 levels: *full* vs. *truncated*).

**Table 2:** Example of materials used in Experiment 1. Orthogonal manipulation of *Passive-Type* (2 levels: *be* vs. *get*) and *Adjunct Type* (2 levels: *full* vs. *truncated*).


be	truncated	The composer was seduced
	Full	The composer was seduced by the dancer

get	truncated	The composer got seduced
	Full	The composer got seduced by the dancer


The main verbs used in the materials were selected based on having a balanced likelihood of appearing in both be-passives and get-passives. This balance was determined using frequency data from both the British National Corpus (BNC) and the Corpus of Contemporary America English (COCA). As noted in the Introduction, passivization can occur for a variety of reasons, such as agent-patient contrasts in discourse topicality or animacy. To avoid influence from such factors, all materials were presented without preceding discourse and all agents and patients were human (i.e., animate) protagonists. A full list of materials is provided in the Appendix (Table A1).

Eighteen filler sentences were also included in the stimulus set. All fillers were grammatical and in active voice. However, they varied structurally and were designed to distract participants from the intentions of the study. The fillers provided a wide range of potential candidates for participants to change. These included semantically implausible situations (e.g., *The milkman read the lamp*), unusual or unexpected collocations (e.g., *The assassin danced through the hallway*), and altered versions of established phrases and idioms (e.g., *He looked wide and far*). Given these particularly salient features suitable for change, it appears unlikely that participants would suspect any common factor (such as formality of one form over another) as being the intended change sought by the experimenters.

### Procedure

In all experiments presented here, a rating paradigm was combined with a subsequent free-response option. In Experiment 1, the sixteen experimental items were randomly divided into two sets of eight items each, such that each participant saw one half of the total of 16 experimental items. This was to minimise the duration of the procedure since it was carried out online (and without payment), ensuring participants would remain attentive throughout.

For each of the two random sets of materials, the 8 (items) × 4 (conditions) were assigned to four separate lists such that each item appeared once per list, and in a different condition in each of the four lists. This resulted in a total of eight lists; four lists for one half of the experimental materials and four for the other half. There were two experimental items per condition per list, plus all 18 filler items, giving 26 sentences in total per list.

**Figure 1:**
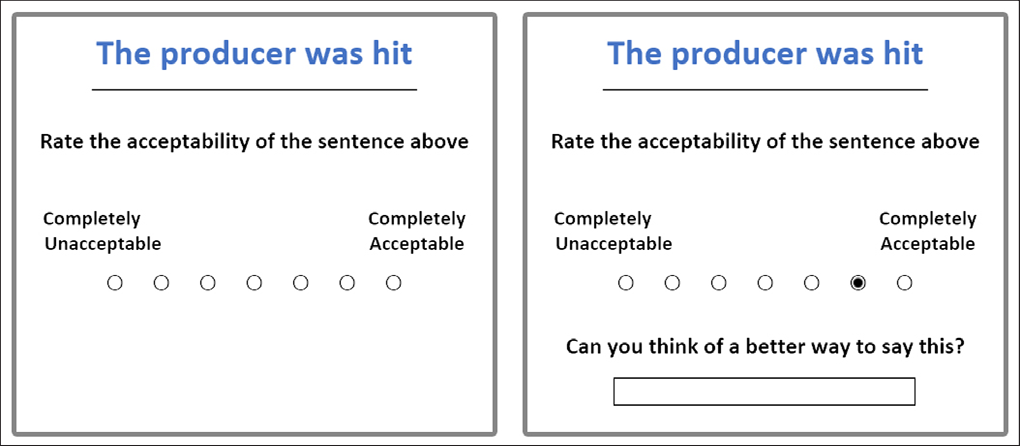
Illustration of trial procedure.

To take part, participants logged in to the host website and selected this experiment from a list of studies. When registering on the website, data such as age and gender are required, allowing these variables to be monitored. After selecting the experiment, participants were randomly assigned to one of the eight lists and were presented with brief instructions before commencing the experiment. Trials appeared in a pseudo-random order, each proceeding as in Figure 1. A sentence was given at the top of the screen, which participants were asked to rate on an unnumbered, seven-point scale below. Text labels at the poles of the scale indicated a range from ‘completely unacceptable’ to ‘completely acceptable’. It is important to note that all the sentences considered here (as well as in Experiments 2 and 3) were perfectly grammatical. Nevertheless, ratings of acceptability were used in order to get at the relative preference for each form while avoiding any need for explicit comparison, since such an approach would draw direct attention to the purpose of the study. After selecting a rating, a text field appeared below with the question *Can you think of a better way to say this?* Participants were free to type a suggestion or to leave the field blank. Once they had finished, they clicked an on-screen button to begin the following trial.

At the end of the session there was a brief note thanking them for participation and contact details if they had any questions about the experiment. A typical experimental session lasted about 5–10 minutes.

### Data analysis

The acceptability ratings, suggestion likelihoods, and type of suggestion counts from this and the following experiments^4^ were first aggregated into subject-by-condition means (or totals), and item-by-condition means (or totals) respectively. The aggregated data were then analysed using non-parametric bootstrapping (10,000 resamples) to derive 95% confidence intervals across subjects and items, respectively (see, e.g., Davison & Hinkley, 1997). Care must be taken to ensure that repeated-measures dependencies in the original data are preserved within the bootstrap samples. We therefore treated data provided by the same subject (respectively item) as one unit for resampling.

### Experiment 1 Results

#### Acceptability ratings

**Figure 2:**
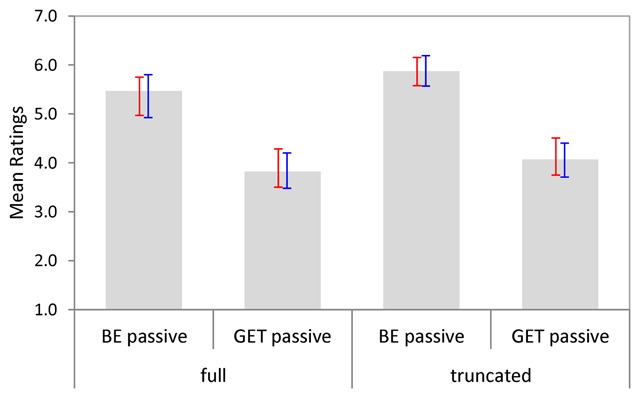
Mean acceptability ratings per condition (Experiment 1). Bootstrapped 95% CIs are shown by participants (red) and items (blue).

Figure 2 shows the average acceptability ratings per condition, with bootstrapped 95% CIs by subjects and items. As can be seen, be-passives were rated as more acceptable than get-passives overall. Also, there was a small numerical trend towards higher acceptability ratings for truncated passives as compared to full passives.

Ninety-five percent confidence intervals for cross-condition differences established a reliable main effect of Passive Type only (Table 3). The main effect of Adjunct Type was reliable by participants but not by items, and the interaction did not approach significance.

**Table 3:** Bootstrapped 95% CIs [*lower limit, upper limit*] for cross-condition differences relating to the main effect of Passive Type, the main effect of Adjunct Type, and the Passive Type × Adjunct Type interaction in the acceptability ratings from Experiment 1. Significant effects (CIs not enclosing zero) are highlighted with an asterisk.

Effect	Test Contrast	By Subjects		By Items	

Passive Type (P)	*avg*(BE)–*avg*(GET)	[1.23, 2.00]	*	[1.33, 2.05]	*
Adjunct Type (A)	*avg*(full)–*avg*(trunc)	[–0.62, –0.14]	*	[–0.78, 0.06]	
P × A interaction	(fullBE–fullGET)–(truncBE–truncGET)	[–0.79, 0.23]		[–0.75, 0.30]	

#### Suggestion likelihood

**Figure 3:**
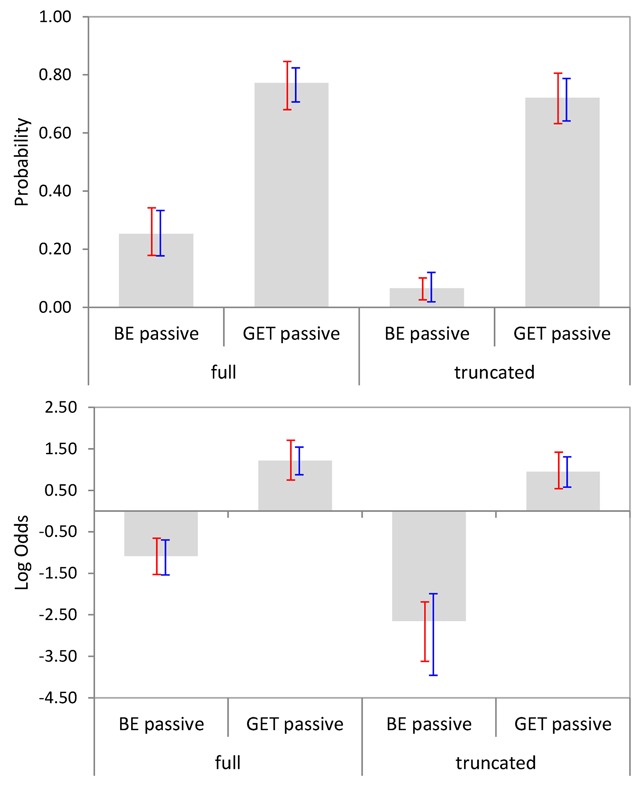
Mean suggestion likelihoods per condition (Experiment 1). Bootstrapped 95% CIs are shown by participants (red) and items (blue). The upper panel shows raw probabilities and the lower panel shows data projected on a *log odds* scale.

We also considered how likely participants were to suggest a change for each sentence. For this stage of analysis, we included only those responses that implied a change to a transitive variant (other types of change, such as substituting a synonym or a related word, constituted fewer than 10% of the suggestions per condition and were not considered). Figure 3 shows the relevant means with bootstrapped 95% CIs per condition, both in raw probabilities (upper panels) and on a *log odds* scale (lower panels). Note that a probability of .5 corresponds to 0 on the log odds scale. As can be seen, be-passives were far less likely to be changed than get-passives, particularly when no agentive by-phrase occurred in the sentence.

Ninety-five percent confidence intervals for cross-condition differences (*log odds* data only) showed that there was a reliable main effect of Passive Type (with get-passives more likely to be changed than be-passives), a reliable main effect of Adjunct Type (with full passives more likely to be changed than truncated passives), and finally, a significant interaction between the two factors, with the effect of Passive Type being more pronounced for truncated passives, see Table 4. The 95% CIs in Figure 3 indicate that the interaction was mainly due to an increase in the likelihood of changing be-passives when the latter included an agentive by-phrase.

**Table 4:** Bootstrapped 95% CIs [*lower limit, upper limit*] for cross-condition differences relating to the main effect of Passive Type, the main effect of Adjunct Type, and the Passive Type × Adjunct Type interaction in the *log odds* of making a suggestion in Experiment 1. Significant effects (CIs not enclosing zero) are highlighted with an asterisk.

Effect	Test Contrast	By Subjects	By Items

Passive Type (P)	*avg*(BE)–*avg*(GET)	[–3.54, –2.53]*	[–3.86, –2.32]*
Adjunct Type (A)	*avg*(full)–*avg*(trunc)	[0.56, 1.43]*	[0.56, 1.56]*
P × A interaction	(fullBE–fullGET)–(truncBE–truncGET)	[0.59, 2.53]*	[0.62, 2.47]*

#### Types of suggestions

**Figure 4:**
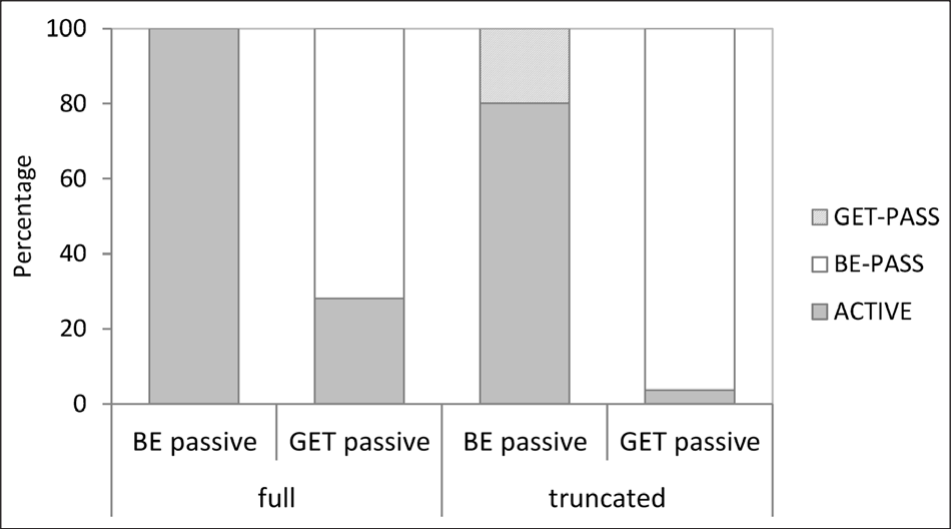
Distribution of the types of suggestions made in Experiment 1, shown as percentages out of the total number of suggestions per condition.

Related to the previous analysis, we examined the types of suggestions made in each condition. In this more descriptive analysis, we focused on numbers of changes from be-passive to get-passive (or active voice, respectively), and numbers of changes from get-passive to be-passive (or active voice, respectively). Figure 4 shows the relevant data as percentages out of the total number of suggestions made in each condition. Bootstrapped 95% CIs for the corresponding raw counts are shown in Table 5.

**Table 5:** Bootstrapped 95% CIs [*lower limit, upper limit*] for raw counts of suggestions made in Experiment 1, broken down by Passive Type of the original sentence (be vs. get), Adjunct Type in the original sentence (full vs. truncated) and type of suggestion made (Active, Be-passive, or Get-passive).

		Suggestion (95% CI by Subjects)			Suggestion (95% CI by Items)		

Passive Type	Adjunct Type	Active	Be-passive	Get-passive	Active	Be-passive	Get-passive

be	full	[27, 52]	–	[0, 0]	[27, 51]	–	[0, 0]
	truncated	[3, 14]	–	[0, 5]	[2, 15]	–	[0, 5]

get	full	[22, 48]	[71, 103]	–	[23, 45]	[73, 101]	–
	truncated	[0, 9]	[90, 120]	–	[0, 9]	[88, 121]	–

As becomes evident, be-passives were almost exclusively changed into active voice sentences, although a very small number (statistically indistinguishable from zero) of changes to get-passive did occur in the truncated be-passive condition. In stark contrast, get-passives were considerably more likely to be changed into be-passives than into active voice sentences, particularly when the original get-passive sentence did not include an agentive by-phrase – in this latter case, numbers of suggested changes into active voice were actually not significantly different from zero. On the few occasions a truncated passive was changed into an active, a generic phrase such as ‘someone’ was used in subject position.

#### Summary

As expected, be-passives were rated as more acceptable than get-passives. We also found that changes were more likely when passives contained an agentive by-phrase. The latter became specifically apparent in changes made to be-passives, with the presence of an agentive by-phrase facilitating their change into active voice.

Most strikingly, participants displayed an overwhelming tendency to change be-passives into active voice, yet get-passives were predominantly changed into be-passives rather than active voice. While this pattern was predicted by the general descriptions in Thompson et al. (2013), the strength of these preferences is nonetheless remarkable, highlighting differences in representational overlap between be-passive and active voice versus get-passive and active voice.

## Experiment 2

In Experiment 2 we expanded on the findings of Experiment 1 by examining two further adjunct types in addition to the agentive by-phrase used in Experiment 1. Using these three adjunct types, we examined the effects of the presence versus absence of an agent, and the presence versus absence of the preposition ‘by’. In Experiment 2 we also utilised a more sensitive rating scale to be able to capture more subtle differences between conditions.

### Participants

A new sample of sixty native-English speakers (age 19–64, mean age 26; 32% males) were tested in individual laboratory sessions. They received subject payment or course credits for their participation. As before, participants were either undergraduate students at the University of Glasgow, or recruited through the university’s subject database. Participants gave informed consent, and were free to withdraw at any time.

### Stimuli

Eighteen sets of materials were created based on those in Experiment 1, though in this case there were six conditional variants (Table 6). Each item was a single sentence in passive voice. As before, sentences were formed using either *be* or *get*, which constituted the first conditional manipulation: *Passive-Type*. The second conditional manipulation was *Adjunct-Type*, with three variants: Each sentence included either an *agentive by-phrase* (as in Experiment 1), a *non-agentive by-phrase*, or a *non-by adjunct*. This resulted in a 2 (Passive-Type levels) × 3 (Adjunct-Type levels) within-subjects and within-items design. A full list of materials is provided in the appendix (Table A2).

**Table 6:** Example of materials used in Experiment 2 with orthogonal manipulation of *Passive-Type* (2 levels: *be* vs. *get*) and *Adjunct-Type* (3 levels: *agentive by-phrase* vs. *non-agentive by-phrase* vs. *non-by adjunct*).


be	agentive by-phrase	The composer was seduced by the dancer
	non-agentive by-phrase	The composer was seduced by the end of the session
	non-by adjunct	The composer was seduced in a dark back lane

get	agentive by-phrase	The composer got seduced by the dancer
	non-agentive by-phrase	The composer got seduced by the end of the session
	non-by adjunct	The composer got seduced in a dark back lane


### Procedure

The procedure largely followed that of Experiment 1. Using a PC running custom-made software based on Flash Player, participants were presented with a single sentence in each trial; they rated it, and were given the option of suggesting a ‘better way’ to say it. The slight alterations for Experiment 2 were that it was run in the lab rather than online, and in place of a seven-point Likert scale (cf. Experiment 1) we provided a line with a continuous slider (with extreme poles indicating a scale from 0 to 100). Each session lasted around 25 minutes.

The 18 (items) × 6 (conditions) were assigned to six separate lists such that each item appeared once per list, in a different condition in each of the six lists. There were three items per condition per list, ensuring an equal frequency of each condition in each list. Pseudo-randomly interspersed with the 18 critical items per list, there were 38 filler items comparable to those of Experiment 1. This gave a total of 56 trials per experimental session.

### Experiment 2 Results

#### Acceptability ratings

**Figure 5:**
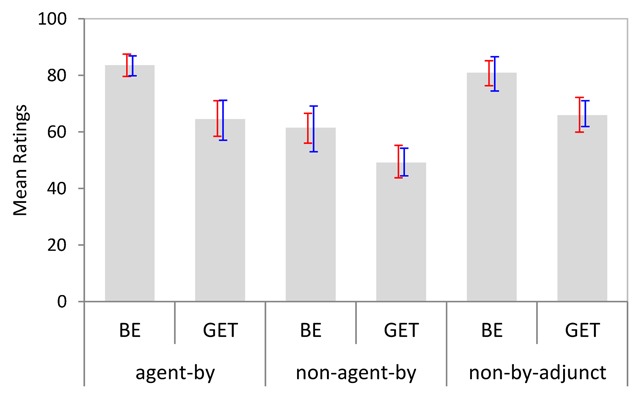
Mean acceptability ratings per condition (Experiment 2). Bootstrapped 95% CIs are shown by participants (red) and items (blue).

Figure 5 shows the average acceptability ratings per condition, with bootstrapped 95% CIs by subjects and items. Again, be-passives were generally found to be more acceptable than get-passives. Also, acceptability ratings were generally lower for sentences containing non-agentive by-phrases than for sentences containing agentive by-phrases or non-by adjuncts.

Bootstrapped 95% confidence intervals for cross-condition contrasts (Table 7) confirmed these descriptive observations by establishing a reliable main effect of Passive Type, a reliable main effect of Adjunct Type, and no significant interaction between the two factors. Note that effects involving Adjunct Type have two effect degrees of freedom, which is why two 95% CIs are reported for the Adjunct Type main effect as well as for the Passive Type × Adjunct Type interaction: one for the contrast between the *agentive by-phrase* and the *non-agentive by-phrase* condition, and one for the contrast between the *agentive by-phrase* and the *non-by adjunct* condition.

**Table 7:** Bootstrapped 95% CIs [*lower limit, upper limit*] for cross-condition differences relating to the main effect of Passive Type, the main effect of Adjunct Type, and the Passive Type × Adjunct Type interaction in the acceptability ratings from Experiment 2. Significant effects (CIs not enclosing zero) are highlighted with an asterisk; ‘a’ = *agent-by*; ‘na’ = *non-agent-by*; ‘nb’ = *non-by-adjunct*.

Effect	Test Contrast	By Subjects		By Items	

Passive Type (P)	*avg*(BE)–*avg*(GET)	[10.8, 19.9]	*	[12.2, 18.3]	*
Adjunct Type (A)	*avg*(a)–*avg*(na)	[14.2, 23.6]	*	[11.2, 26.0]	*
	*avg*(a)–*avg*(nb)	[–3.5, 4.7]		[–5.7, 5.9]	
P × A interaction	(aBE–aGET) – (naBE–naGET)	[–1.5, 15.5]		[–0.9, 15.9]	
	(aBE–aGET) – (nbBE–nbGET)	[–2.4, 10.6]		[–5.4, 15.0]	

#### Suggestion likelihood

Again, we considered how likely participants were to suggest a change for each sentence, including only those responses that implied a change to another transitive variant of the sentence (other types of changes, such as replacing content words, constituted fewer than 10% of the suggestions in each condition).

**Figure 6:**
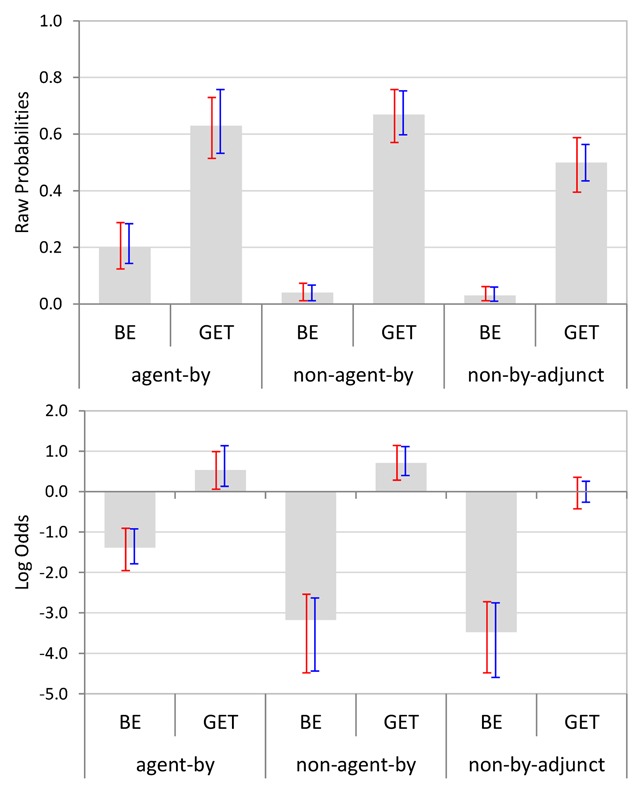
Mean suggestion likelihoods per condition (Experiment 2). Bootstrapped 95% CIs are shown by participants (red) and items (blue). The upper panel shows raw probabilities and the lower panel shows data projected on a *log odds* scale.

Figure 6 shows the relevant means with bootstrapped 95% CIs per condition, both in raw probabilities (upper panel) and on a *log odds* scale (lower panel). As in Experiment 1, be-passives were far less likely to be changed than get-passives, particularly when no agentive by-phrase occurred in the sentence. As shown by the bootstrapped 95% confidence intervals for cross-condition contrasts (*log odds* data only) in Table 8, there was a reliable main effect of Passive Type, a reliable main effect of Adjunct Type (suggestion likelihoods were generally higher in the agentive by-phrase condition than in either of the other two Adjunct Type conditions), and a significant interaction between Passive Type and Adjunct Type: Relative to the agentive by-phrase condition, the get-passive versus be-passive contrast in suggestion likelihood was proportionally larger in both the non-agentive by-phrase condition and in the non-by adjunct condition. As can be seen from the 95% CIs around the means in Figure 6, the Adjunct Type main effect as well as the interaction were mostly driven by a substantial increase in the likelihood of changing (specifically) be-passives when they were presented with an agentive by-phrase.

**Table 8:** Bootstrapped 95% CIs [*lower limit, upper limit*] for cross-condition differences relating to the main effect of Passive Type, the main effect of Adjunct Type, and the Passive Type × Adjunct Type interaction in the *log odds* of making a suggestion in Experiment 2. Significant effects (CIs not enclosing zero) are highlighted with an asterisk; ‘a’ = *agent-by*; ‘na’ = *non-agent-by*; ‘nb’ = *non-by-adjunct*.

Effect	Test Contrast	By Subjects	By Items

Passive Type (P)	*avg*(BE)–*avg*(GET)	[–3.71, –2.60]*	[–3.78, –2.64]*
Adjunct Type (A)	*avg*(a)–*avg*(na)	[0.34, 1.46]*	[0.52, 1.38]*
	*avg*(a)–*avg*(nb)	[0.78, 1.93]*	[0.81, 2.05]*
P × A interaction	(aBE–aGET) – (naBE–naGET)	[1.17, 3.26]*	[1.25, 3.14]*
	(aBE–aGET) – (nbBE–nbGET)	[0.58, 2.70]*	[0.43, 3.00]*

#### Types of suggestions

**Figure 7:**
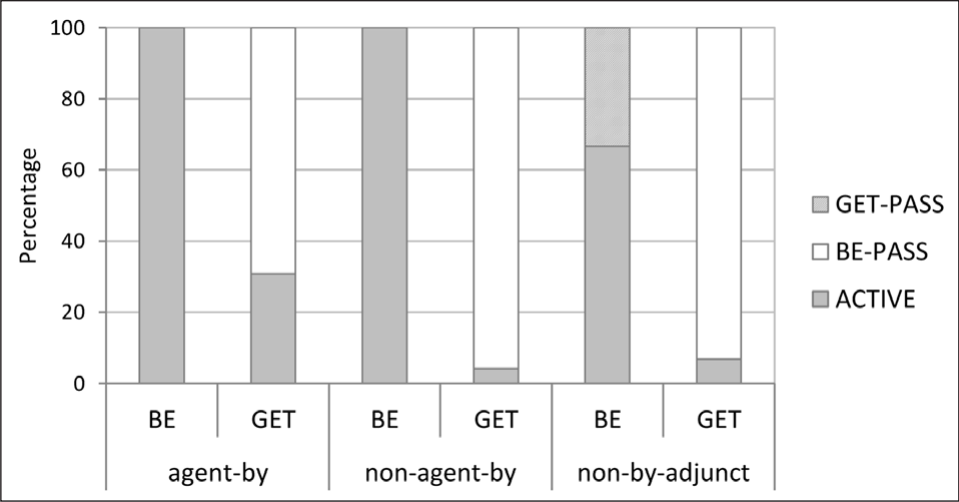
Distribution of the types of suggestions made in Experiment 2, shown as percentages out of the total number of suggestions per condition.

Next, we examined the types of transitive-description changes made in each condition; that is, changes from be-passive to get-passive (or active voice) and changes from get-passive to be-passive (or active voice). Figure 7 shows the relevant distribution (as percentages out of the total number of suggestions made per condition) and Table 9 reports bootstrapped 95% CIs for the corresponding raw counts.

**Table 9:** Bootstrapped 95% CIs [*lower limit, upper limit*] for raw counts of suggestions made in Experiment 2, broken down by passive type of the original sentence (be vs. get), type of adjunct in the original sentence (agent-by vs. non-agent-by vs. non-by) and type of suggestion made (Active, Be-passive, or Get-passive).

		Suggestion (95% CIs by Subjects)			Suggestion (95% CIs by Items)		

Passive Type	Adjunct Type	Active	Be-passive	Get-passive	Active	Be-passive	Get-passive

be	agent-by	[22, 51]	–	[0, 0]	[25, 47]	–	[0, 0]
	non-agent-by	[2, 13]	–	[0, 0]	[3, 12]	–	[0, 0]
	non-by	[1, 8]	–	[0, 5]	[1, 8]	–	[0, 5]

get	agent-by	[20, 49]	[59, 94]	–	[22, 49]	[60, 92]	–
	non-agent-by	[1, 10]	[96, 129]	–	[0, 12]	[98, 126]	–
	non-by	[2, 11]	[63, 98]	–	[2, 11]	[70, 92]	–

The observed response patterns were highly comparable to those in Experiment 1: be-passives were nearly always changed into active voice sentences, except for a very small number (statistically indistinguishable from zero) of suggested get-passive changes when the be-passive had a non-by adjunct. Get-passives, however, were considerably more likely to be changed into be-passives than into active voice sentences, and again, this preference for change into be-passive was considerably more pronounced when the original get-passive sentence did not include an agentive by-phrase (i.e., a non-agentive by-phrase or a non-by adjunct).

## Experiment 3

In the two experiments described above, we asked participants to rate ‘acceptability’ and gave the opportunity to suggest a ‘better way’ to say each sentence. A concern that can be raised is that the observed effects might be a matter of normative corrections induced by the wording of our instructions. However, this does not have any apparent bearing on the tendency to make changes in single steps; one would still expect participants to leap directly to the most prescriptively ‘correct’ form (i.e., active voice). We therefore assume that the reason for the observed one-step-at-a-time phenomenon is related to processing, and should be present regardless of the specific reason participants are asked to seek alternative sentences.

Nonetheless, it is important to consider the impact of using alternative instructions, to establish the generality of our effects. As noted in the Introduction, the relative preference for each form may induce some directionality to the types of changes made; that is, from less to more acceptable. Without this focus, the directionality may disappear, allowing be-passives to be changed both ‘upwards’ into active voice (as in the previous experiments) and ‘downwards’ into the less preferred get-passive form. At the same time, we still expect to see changes happening in single steps.

In Experiment 3 we used the materials from Experiment 1 and repeated the procedure with modified instructions, allowing us to establish whether the previous effects were limited to a task that potentially induced a focus on normative correctness.

### Participants

A new sample of forty-eight native-English speakers (age 18–22, mean age 19; 13% males) were tested in individual laboratory sessions in return for course credits. One participant’s data set had to be excluded because of a technical fault. Participants were mainly undergraduate students at the University of Nottingham, and all gave informed consent and were free to withdraw at any time.

### Stimuli

The sixteen sets of materials used in Experiment 1 were used again here (see Table 2); only the task instructions were altered. As such, Experiment 3 had a 2 (Passive-Type) × 2 (Adjunct-Type) within-subjects, within-items design. Another minor change from Experiment 1 was that each participant now saw all 16 items (four per condition). That is, experimental materials were not split into subgroups of eight as in Experiment 1.

### Procedure

The procedure closely followed that of the first experiment: participants were presented with a single sentence in each trial, which they rated, and could then optionally suggest an alternative. However, rather than rating *acceptability* on a scale from ‘completely unacceptable’ to ‘completely acceptable’, they instead were asked ‘How do you find this sentence?’, with a scale running from ‘very unusual’ to ‘completely normal’. When provided with the opportunity to suggest an alternative, rather than asking for a ‘better way’ to say the sentence, participants were simply asked ‘How would you say this?’. As before, they could leave the suggestion response blank.

In contrast to Experiment 1, which was run online with participants only seeing half of the materials each to keep the task brief, this experiment was run in the lab, and so participants saw all materials. The sixteen (items) × 4 (conditions) were assigned to two lists, with each item appearing once per list and in a different condition in each. There were eight experimental items per condition per list, plus 18 filler items, giving 34 sentences per list. Sessions lasted approximately 10 minutes.

### Experiment 3 Results

#### Naturalness ratings

**Figure 8:**
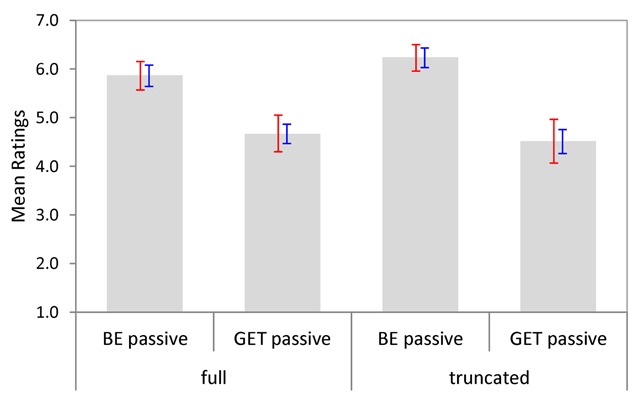
Mean naturalness ratings per condition (Experiment 3). Bootstrapped 95% CIs are shown by participants (red) and items (blue).

Figure 8 shows the average naturalness ratings per condition, with bootstrapped 95% CIs by subjects and items. As can be seen, be-passives were (again) rated as more acceptable than get-passives overall.

Ninety-five percent confidence intervals for cross-condition differences established a significant main effect of Passive Type and a significant Passive Type × Adjunct Type interaction; the main effect of Adjunct Type did not approach significance (see Table 10). The interaction was due to a reliably more pronounced Passive Type simple effect when the original sentence contained no agentive by-phrase, and Figure 8 further suggests that mentioning the agent in the original sentence (full passive) was slightly more detrimental to the naturalness of be-passives than to the naturalness of get-passives.

**Table 10:** Bootstrapped 95% CIs [*lower limit, upper limit*] for cross-condition differences relating to the main effect of Passive Type, the main effect of Adjunct Type, and the Passive Type × Adjunct Type interaction in the acceptability ratings from Experiment 3. Significant effects (CIs not enclosing zero) are highlighted with an asterisk.

Effect	Test Contrast	By Subjects		By Items	

Passive Type (P)	*avg*(BE)–*avg*(GET)	[1.13, 1.80]	*	[1.26, 1.66]	*
Adjunct Type (A)	*avg*(full)–*avg*(trunc)	[–0.29, 0.08]		[–0.32, 0.12]	
P × A interaction	(fullBE–fullGET)–(truncBE–truncGET)	[–0.96, –0.10]	*	[–0.83, –0.23]	*

#### Suggestion likelihood

**Figure 9:**
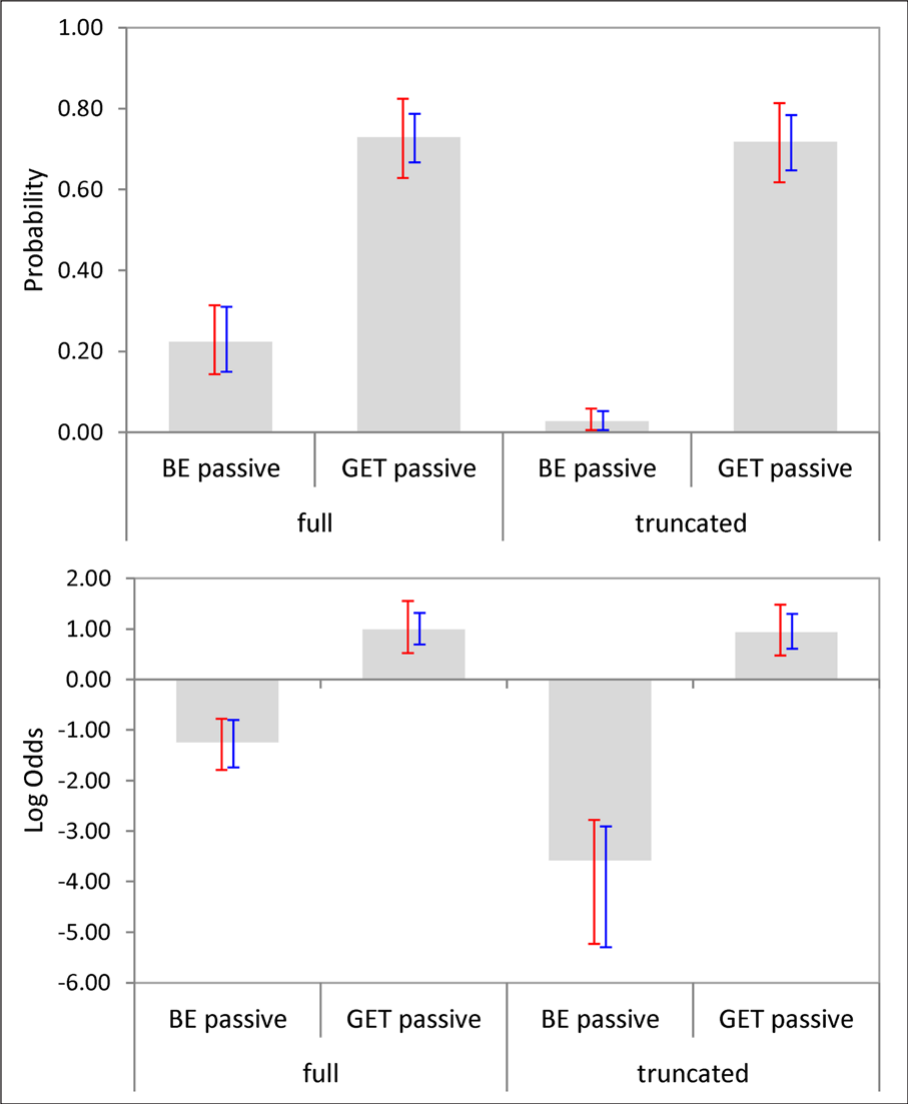
Mean suggestion likelihoods per condition (Experiment 3), with bootstrapped 95% CIs by participants (red) and items (blue). The upper panel shows raw probabilities and the lower panel shows data projected on a *log odds* scale.

Next, we considered how likely participants were to suggest a change for each sentence. As in the previous studies, we included only responses that implied a change to a transitive variant (other types of change constituted fewer than 5% of the suggestions per condition). Figure 9 shows the relevant means with bootstrapped 95% CIs per condition, both in raw probabilities (upper panel) and on a *log odds* scale (lower panel). As in the previous two experiments, be-passives were far less likely to be changed than get-passives, particularly when no agentive by-phrase occurred in the sentence.

Ninety-five percent confidence intervals for cross-condition differences (*log odds* data only) showed that there was a reliable main effect of Passive Type (with get-passives more likely to be changed than be-passives), a reliable main effect of Adjunct Type (with full passives more likely to be changed than truncated passives), and a significant interaction between the two factors, with the effect of Passive Type being more pronounced for truncated passives, see Table 11. The 95% CIs in Figure 9 indicate that the interaction was mainly due to an increase in the likelihood of changing be-passives when the latter included an agentive by-phrase – very much in line with the previous two experiments.

**Table 11:** Bootstrapped 95% CIs [*lower limit, upper limit*] for cross-condition differences relating to the main effect of Passive Type, the main effect of Adjunct Type, and the Passive Type × Adjunct Type interaction in the *log odds* of making a suggestion in Experiment 3. Significant effects (CIs not enclosing zero) are highlighted with an asterisk.

Effect	Test Contrast	By Subjects	By Items

Passive Type (P)	*avg*(BE)–*avg*(GET)	[–4.37, –2.74]*	[–4.30, –2.88]*
Adjunct Type (A)	*avg*(full)–*avg*(trunc)	[0.72, 1.98]*	[0.69, 1.96]*
P × A interaction	(fullBE–fullGET)–(truncBE–truncGET)	[1.32, 3.83]*	[1.46, 4.00]*

#### Types of suggestions

**Figure 10:**
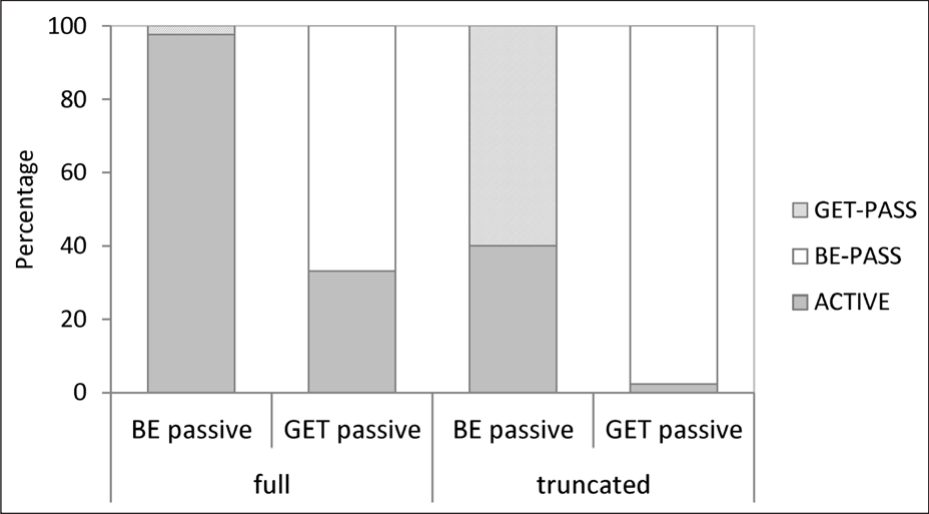
Distribution of the types of suggestions made in Experiment 3, shown as percentages out of the total number of suggestions per condition.

Finally, we examined the types of suggestions made in each condition, focusing on numbers of changes from be-passive to get-passive (or active voice, respectively), and numbers of changes from get-passive to be-passive (or active voice, respectively). Figure 10 shows the relevant data as percentages out of the total number of suggestions made per condition. Bootstrapped 95% CIs for the corresponding raw counts are shown in Table 12.

**Table 12:** Bootstrapped 95% CIs [*lower limit, upper limit*] for raw counts of suggestions made in Experiment 3, broken down by Passive Type of the original sentence (be vs. get), Adjunct Type in the original sentence (full vs. truncated) and type of suggestion made (Active, Be-passive, or Get-passive).

		Suggestion (95% CI by Subjects)			Suggestion (95% CI by Items)		

Passive Type	Adjunct Type	Active	Be-passive	Get-passive	Active	Be-passive	Get-passive

Be	full	[26, 58]	–	[0, 3]	[28, 55]	–	[0, 3]
	truncated	[0, 5]	–	[0, 8]	[0, 5]	–	[0, 6]
Get	full	[33, 58]	[72, 110]	–	[36, 54]	[81, 103]	–
	truncated	[0, 7]	[110, 146]	–	[0, 8]	[112, 143]	–

As can be seen, be-passives (particularly when including an agentive by-phrase) were almost exclusively changed into active voice sentences. Changes from be-passive to get-passive were very rare overall (indeed, their occurrences were statistically indistinguishable from zero, see Table 12). Curiously, the percentages in Figure 10 suggest more get-passive than active voice changes in the truncated be-passive condition, which appears to contrast with the findings from the previous two experiments (see Figures 4 and 7). However, given that the present experiment showed a total of only five suggested changes in the truncated be-passive condition, the percentages for this particular condition in Figure 10 should be treated with extreme caution.

In contrast to the pattern for be-passives, get-passives were considerably more likely to be changed into be-passives than into active voice sentences both when the original get-passive sentence included an agentive by-phrase and (even more so) when the original get-passive sentence was truncated; in this latter case, the number of suggested changes into active voice was not appreciably different from zero.

Overall, Experiment 3 makes it evident that having less of a focus on ‘normative correctness’ in the experimental instructions does not result in markedly different response patterns compared to the previous experiments. This replication clearly highlights the robustness of the effects outlined above.

## General Discussion

In psycholinguistics, research on structural choice has largely focussed on the contrast between active voice and passive voice, with no further distinction between passive types (e.g., Bock, et al., 2007; Ferreira, 2003; Segaert et al., 2012; Whitehurst, Ironsmith, & Goldfein, 1974). However, the two passive sub-categories in English (be-passive and get-passive) differ from each other in precisely the ways that are the focus of the structural choice literature, such as affectedness or agency. Here, we addressed this limitation in the literature by examining the two passive types independently.

In more theoretical terms, numerous proposals have been put forward to describe and explain syntactic and semantic differences between the get-passive and the be-passive. A lack of agreement in the current literature can partly be blamed on insufficient empirical research. Our aim here was to investigate and provide empirical evidence of how the three transitive variants (be-passive, get-passive, and active voice) are mentally represented in relation to each other, providing insight into how similar the underlying structures of each should be assumed to be. We also considered the additional influence of various types of adjunct in assessing the similarities of these three variants. The resulting findings should be of interest to both linguistic and psycholinguistic theories of passivization and to language production, syntax, and semantics generally.

We conducted two studies in which participants rated the acceptability of passive sentences, and were given an opportunity to change those sentences into ‘a better way of saying’ them if they deemed it appropriate. Each sentence was formed either with *be* or with *get*. In Experiment 1, the passives either included an agentive by-phrase or were truncated (included no adjunct). In Experiment 2, the passives included one of three adjunct types: an agentive by-phrase (as in Experiment 1); a non-agentive by-phrase; or a non-by adjunct. We also conducted a third study in which we considered the possible impact of having instructions that focused on ‘naturalness’ and ‘a different way to express the sentences’, rather than on ‘acceptability’ and ‘a better way to express the sentences’. Both types of instruction were intended to partially constrain the range of responses from participants, such that suggested alternatives would be equivalent in meaning. As discussed in the literature, there are a variety of other syntactic structures that are related to the passive constructions such as the causative (Huang, 1999; Fleisher, 2008; Thompson & Scheepers, 2013) and ‘middle’ constructions (Keyser & Roeper, 1984; Alexiadou & Doron, 2012). In order to investigate the relationship between the two passive-types and additional related structures, a more open-ended task could be used.

Following Thompson et al. (2013), we assume that the three transitive variants can be uniquely described by the interaction of two dimensions: *Patient Importance* (relating to how much emphasis is on the patient) and *Patient Prominence* (relating to whether the patient assumes the grammatical subject role in the sentence). These are taken to be the primary factors distinguishing the transitive variants, and were the main focus of the studies in the current paper. However, we assume that additional aspects of structure modulate interpretation and usage. As such, we also considered the impact of several types of adjunct.

Our main predictions were that get-passives would be preferentially changed into be-passives, and be-passives would be preferentially changed into active voice, since it should be easier to enact a change requiring only one dimension to be altered than a change requiring two dimensions to be altered. That is, we expected greater *representational similarity* to allow for easier changes. These predictions were largely confirmed by our data, and there was no suggestion that type of instruction (focusing more or less on normative correctness in responding) would strongly modulate the outcome.

We also expected be-passives to be rated as more acceptable than get-passives, in line with corpus frequencies. Likewise, we expected truncated passives to be preferred over full passives. More importantly, we also predicted that the inclusion of an agentive by-phrase would make changes into active voice easier. With regards to the latter, we further distinguished between two more refined hypotheses, related to the strength of the effect of representational similarity: if the effect of representational similarity is strong, we would expect an interaction between Truncation and Passive-type (with the likelihood of changing be-passives being more strongly affected by the presence/absence of an agentive by-phrase than the likelihood of changing get-passives); if the effect of representational similarity is weak, we would expect to see a main effect of Truncation only. Our data appeared more in line with a strong influence of representational similarity. In what follows, we provide a more detailed discussion of the individual findings.

### Transitive variants

The most significant finding was that when alternative suggestions were provided, their distribution followed the pattern predicted by representational similarity in Thompson et al.’s (2013) general description. When suggestions were given for get-passives, participants changed them overwhelmingly frequently into be-passives (at a rate of around 85% across experiments and adjunct conditions). Though they received fewer suggestions in total, when be-passives were given suggested alternatives, they were preferentially changed into active voice (at a rate of over 95%). These preferences were consistently present across all experiments, and across all adjunct types examined. It is important to keep in mind that the findings in Thompson et al. (2013) were based on paraphrases of original (context-embedded) active voice sentences into either be-passives or get-passives. Here, we examined paraphrases of original get- versus be-passive sentences into active voice (or each other’s alternative form, respectively) and found that the distribution of responses can be described in terms of the same representational similarity dimensions (Patient Prominence and Patient Importance) whereby active voice is representationally closer to the be-passive than to the get-passive.

As expected, acceptability ratings correlated with corpus frequencies: be-passives were rated as more acceptable overall than get-passives. This was also reflected in suggestion likelihoods, with get-passives receiving suggestions more frequently than be-passives. On a more general level, our data appear to confirm established findings whereby active voice is the most preferred and frequently used form, followed by the be-passive, and finally the get-passive. These frequencies can be observed in various corpora (e.g. BNC, COCA), and in earlier research (e.g. Chafe, 1982; Mair & Leech, 2006).

We also expected that changes from the (less common) get-passive into the (more common) be-passive would be more likely than changes in the opposite direction; that is, changes would be somewhat influenced by frequency of occurrence of each form of transitive description. It is important to note, however, that frequency of occurrence can only explain the directionality and not the ‘stepwise’ nature of these changes. Indeed, when considering a get-passive, the option that would maximise frequency of occurrence would be a direct change into active voice (which, in general usage, is more frequent and preferred than even the be-passive); however, while such get-passive to active voice changes did occur, they were significantly outnumbered by changes from get-passive into the representationally more similar be-passive. Representational similarity therefore still appears to play a defining role in changes between transitive variants.

Rather than frequency of occurrence, semantic differences between the two passive forms could offer an interesting alternative explanation of why changes from get-passive to be-passive were more readily produced than changes in the opposite direction. While *be* is always taken to be an auxiliary verb with only the most basic of semantic information, in various approaches passive *get* is taken to be a lexical verb, with some (Haegeman, 1985) or all (Thompson & Scheepers, 2013) of the semantic range inherent in the standard non-passive form of *get*. Since the lexeme *get* has a broader range of meanings than *be*, a sentence formed with *get* has a wider variety of potential meanings and comes with additional assumptions or connotations. These are absent in the be-passive, since *be* is an auxiliary, semantically limited to a logical operation. Following this logic, *get* adds to the semantic content of a sentence, while *be* does little to alter or add to the semantic content. A change from be-passive to get-passive *adds* information or assumptions, while a change from get-passive to be-passive *removes* some. We assume that, in general, the removal of information should also be easier to achieve than the addition or creation of new information. For example, given an original sentence “Jane intentionally annoyed Mike”, and a rephrased version “Jane annoyed Mike”, the latter remains true even though it under-specifies the original. However, given an original sentence “Pete annoyed Sally”, and a rephrased version “Pete intentionally annoyed Sally”, the latter is not necessarily true. Likewise, the addition of semantics in a change from be-passive to get-passive should be more difficult than the removal of semantics in a change from get-passive to be-passive.

### Adjuncts

In Experiment 1, as expected, there was a general preference for truncated passives, which was, however, more clearly visible in suggestion likelihoods (i.e., participants were more likely to change full passives than truncated passives) than in acceptability ratings (i.e., truncated passives were only marginally more acceptable than full passives). Notably, this Truncation main effect in suggestion likelihood was further modulated by a Passive Type by Truncation interaction. Even though full passives were more likely to be changed overall, this effect was clearly more pronounced for be-passives than for get-passives. That is, be-passives tended to be converted into active voice regardless of whether they were truncated or full, but the presence of an agent further facilitated this change into active voice. Hence we see an increase in frequency of *be*-to-active changes for full be-passives. On the other hand, get-passives were preferentially changed into be-passives rather than active voice. And although the presence of an agent facilitated a change into active voice overall, this facilitation failed to override the preference for changing get-passives into be-passives.

These effects and interactions were largely borne out in Experiment 3 (which differed from Experiment 1 only in the wording of the instructions): truncated passives were more likely to receive suggested changes and were also judged more natural (significantly so in this instance); likewise, a corresponding Truncation by Passive-Type interaction was observed. As such, the patterns established in Experiment 1 cannot be said to relate to ‘correctness’.

In Experiment 2, sentences that included either an agentive by-phrase or a non-by adjunct were rated as equally acceptable, and both were notably more acceptable than sentences that included a non-agentive by-phrase. This is likely due to by-phrases being given an agentive interpretation “by default” (Liversedge et al., 1998), potentially leading to the impression of an implausible sentence in at least some instances of the *non-agentive by-phrase* condition.

Interestingly, the likelihood of suggestions followed a different pattern, with (particularly) be-passive sentences that included an agentive by-phrase being more likely to receive a suggestion than be-passive sentences with either of the other two adjunct types. That is, in the case of the be-passive, despite sentences with a *non-agentive by-phrase* being less acceptable, it is sentences with an *agentive by-phrase* that are most frequently changed. Combined with Experiment 1 (where full passives were compared with truncated passives, and where a similar increase in suggestion likelihood was observed particularly for full be-passives), this suggests that it is not the presence or absence of a *by-phrase* that triggers more changes of (particularly) be-passives, but indeed the presence or absence of an *agent*. However, this is not the case for get-passives, which are still preferentially changed into be-passives even when an agentive by-phrase is present.

### One step at a time

We can see a clear hierarchy of the three transitive variants: active voice is the most acceptable or preferred, followed by be-passives, and finally get-passive. However, the pattern of changes observed does not support a simple strategy of ‘change to the most preferred form’. It is possible that there is some other single dimension with three levels, along which active voice, be-passive, and get-passive are arranged, though it is unclear what this single factor could be.

Notably, changes follow the pattern predicted by assuming the representational similarities proposed in Thompson et al. (2013): changes are significantly more likely to occur between variants where only one dimension is altered (*get*-to-*be* or *be*-to-active), versus when two dimensions require alteration (as in *get*-to-active). When a passive voice sentence includes an agent mentioned via a by-phrase, the most preferred alternative (active voice) is more readily available than when no agent is present. Despite this added facilitation, and active voice being the most preferred transitive variant, get-passives are still preferentially changed into be-passives. This appears to confirm our predictions for representational similarity exerting a *strong* influence. That is, the representational *dissimilarity* between get-passive and active voice cannot be easily overcome, even by the combination of having a ‘ready-made’ agent and the availability of an even more preferred variant. The result is that participants take *one step at a time*, making a single representational change rather than two, even when making two would produce the most preferred form available.

An analogy can be drawn with Gibson et al. (2013)’s work on noisy-channel models of language comprehension, wherein a given alternative structure will be preferred if it can be achieved via fewer changes than another alternative. They demonstrated that when a listener hears an implausible sentence they are more likely to assume an alternative intended sentence when that alternative has fewer representational differences to the implausible version. The more differences (or changes required) between the two forms, the more likely the implausible version will be interpreted as the speaker’s intention. Although none of the sentences we considered are implausible, they still vary in the number of representational differences, and appear to follow the same logic observed by Gibson et al. (2013); that is, change is facilitated when there are fewer representational differences between forms.

An interesting point of note is that this ‘*one step at a time*’ effect remains true even in the absence of an agent. Without explicit mention of an agent, one might assume that focus is naturally shifted to the patient. This could nullify the Patient Importance dimension, in the sense that the patient may become equally important in both be-passives and get-passives when no agent is mentioned. If this were the case, then in the truncated (agentless) form, both passive types would differ from active voice along a single dimension (Patient Prominence). Since the ‘*one step at a time*’ effect did not diminish in conditions without an agent, we conclude that the dimensions of Patient Importance and Patient Prominence must have *inherent* values in the be-passive and get-passive, meaning these values are not influenced (or are minimally influenced) by the presence or absence of an agent in the sentence.

Given that the two dimensions discussed above are able to distinguish the three transitive variants, we now briefly consider what underlying structural factors may motivate these dimensions. To account for our data, a syntactic theory of the passive should show that the get-passive shares something representationally with the be-passive that is absent in the active voice, while the be-passive should share something representationally with the active voice that it does not share with the get-passive. In Thompson et al. (2013), the Patient Prominence dimension is linked to the assignment, or not, of the Patient to a prominent syntactic role: subject position in English. The authors are less specific about the cause of the Patient Importance dimension beyond the inclusion or exclusion of the lexeme *get*.

Existing representational frameworks for the passive can be broadly classified into raising and control approaches, as briefly outlined in the Introduction. For general raising approaches (Haegeman, 1985; Fleisher, 2008; Alexiadou & Doron, 2012), the thematic patient is raised into subject position in both passive forms, providing a logical motivation for the Patient Prominence alternations. However, there are also notable structural differences between the two (see, e.g., Alexiadou, 2012). A factor that could account for the Patient Importance alternations is the number of lexical verbs: the get-passive has two, while the be-passive and active each have one. This would be dependent on assuming *get* remains a full lexical verb in the passive.

In control approaches (Huang, 1999; Butler & Tsoulas, 2006) the get-passive is assumed to involve a subject control verb, while the be-passive is a raising construction. Such control approaches could also account for Patient Importance alternations via number of lexical verbs. However, Patient Prominence alternations are again problematic, with the be-passive and get-passive having notably distinct structures (one raising, one control). Indeed, under a control approach, it could be argued that the main clause in a get-passive is structurally closer to the *active voice* than to the be-passive, with both the get-passive and active having a directly merged (non-raised) subject. One could expect this to facilitate changes from get-passive to the most preferred active voice form. Given our findings to the contrary, it is difficult to align these structures with the Patient Importance and Patient Prominence dimensions.

While superficially similar, a more recent departure from classic models can been found in the ‘*pvP Theory*’; a distinct type of ‘control’ approach (Thompson & Scheepers, 2013). Briefly, the pvP Theory unites the passive syntax of the be-passive, get-passive, and causative, proposing a small syntactic unit that is present in *all passive types*, and always has the same structure regardless of the passive type. The ‘passiveness’ of a sentence is contained, or generated, entirely within the pvP unit; above this phrase, passives are structurally the same as actives. As such, the main structural difference between active voice and the passive, is the presence versus absence of a pvP phrase. Further, the be-passive and get-passive only differ in the alternation between the verbs *be* and *get*.

This theory leaves both *get* and *be* outside of the pvP structural unit: the two lexemes behave exactly as they do in any other non-passive construction. Therefore no additional explanation is needed for the differing syntax and semantics of be-passives and get-passives, as these differences simply follow from the established natures of *be* and *get*. As *be* is an auxiliary and *get* is a lexical verb, one consequence of this approach is that get-passives have an additional lexical verb (the main verb, plus *get* itself) whereas be-passives have only one lexical verb (the main verb), with *be* being an auxiliary only.

As noted, a ‘pvP unit’ is assumed to be present and identical in all forms of the passive. This means there is one major representational difference between get-passive and be-passive: the number of lexical verbs. Likewise there is one major representational difference between be-passive and active voice (presence versus absence of pvP unit). However, there are *two* representational differences between the get-passive and active voice: the get-passive has an additional lexical verb and contains the pvP unit, which are both absent in active voice. The presence of the pvP unit allows the alternation between [+Patient Prominence] (passive voice) and [–Patient Prominence] (active voice). Crucially, and unlike other approaches, there are no other structural differences that are specific to the passive and absent in the active beyond the pvP unit. As suggested for raising approaches above, we propose that the additional lexical verb that is exclusively present in the get-passive could be responsible for the presence of [+Patient Importance], with its absence resulting in [–Patient Importance].

### Further directions

Here we have focussed on the most significant factors differentiating the mental representations of the two passive-types and the active voice. We can see that these three forms are represented in a hierarchy, such that the be-passive has a more similar representation to active voice than the get-passive does. However, we do not know precisely *how different* each form is from the others.

Examining more fine-grained distinctions between representations of be-passive, get-passive, and active voice, would allow further discussion of the precise underlying structures. Future research could investigate perceptions of semantic similarity, or use a less constraining task in order to consider likelihood of changes between passives and other related structures.

## Conclusion

We have provided empirical data that reveal how the three transitive variants in English (get-passive, be-passive, and active voice) are mentally represented in relation to each other. The pattern of changes observed here are consistent with partial representational overlap along two dimensions: Patient Prominence and Patient Importance (Thompson et al., 2013). Get-passives share the [+Patient Prominence] attribute with be-passives; be-passives share the [–Patient Importance] attribute with active voice; get-passive share neither attribute with active voice. The observed preference for changing get-passives into be-passives, rather than directly into the most preferred form of active voice, is explained by this additional representational difference between the get-passive and active voice.

Given that these representational differences can be conceptualised following the patient-related dimensions of Thompson et al. (2013), we discussed structural factors that could underlie these dimensions. In terms of syntactic structure, raising approaches provide a better fit than control approaches overall. However, the recently proposed *pvP Theory* appears to be the most neatly consistent with the current findings, providing very clear structural parallels to the Patient Prominence and Patient Importance dimensions.

## Additional Files

The additional files for this article can be found as follows:

10.5334/joc.36.s1 Table A1. List of stimuli used in Experiments 1 and 3.

10.5334/joc.36.s1 Table A2. List of stimuli used in Experiment 2.

## Data Availability

The coded data are available via the open repository ‘figshare’ at: https://doi.org/10.6084/m9.figshare.5572663

## Footnotes

Throughout this paper we use the terms ‘Agent’ and ‘Patient’ as short hands for the more accurate generalized role terms ‘Proto-Agent’ and ‘Proto-Patient’ (e.g., Dowty, 1991) or ‘Actor’ and ‘Undergoer’, respectively (e.g., Foley and Van Valin, 1984; Van Valin and La Polla, 1997).

The discussion of syntactic structure included here is rather simplified, since we are currently investigating the broadest distinguishing factors between the passive types. For more in-depth description, see Thompson & Scheepers (2013) or Alexiadou & Shäfer (2013).

The term ‘affected’ is used frequently in the literature (Arrese, 1999; Cameron, 1996; Carter & McCarthy, 1999; Orfitelli, 2011; Sasaki, 1999), but without a consistent definition. It is usually taken to mean roughly that the patient undergoes some manner of change as a result of the action or event.

The coded data from all three studies is available via the open repository figshare, at DOI: https://doi.org/10.6084/m9.figshare.5572663.
