# Supplementary material for: One Step at a Time: Representational Overlap Between Active Voice, Be-passive, and Get-passive Forms in English
Source: J Cogn. 2018 Jun 26;1(1):35. doi: 10.5334/joc.36 (PMC6634362; doi:10.5334/joc.36)
Supplement: Table A1. — List of stimuli used in Experiments 1 and 3. [file joc-1-1-36-s1.pdf]

Appendix

Table A1 List of stimuli used in Experiments 1 and 3.

Item	Condition	Text
1	1	The designer was beaten by the shoemaker
1	2	The designer got beaten by the shoemaker
1	3	The designer was beaten
1	4	The designer got beaten
2	1	The hairdresser was hurt by the tailor
2	2	The hairdresser got hurt by the tailor
2	3	The hairdresser was hurt
2	4	The hairdresser got hurt
3	1	The minister was elected by the mayor
3	2	The minister got elected by the mayor
3	3	The minister was elected
3	4	The minister got elected
4	1	The firefighter was stopped by the assessor
4	2	The firefighter got stopped by the assessor
4	3	The firefighter was stopped
4	4	The firefighter got stopped
5	1	The composer was seduced by the dancer
5	2	The composer got seduced by the dancer
5	3	The composer was seduced
5	4	The composer got seduced
6	1	The banker was trapped by the alchemist
6	2	The banker got trapped by the alchemist
6	3	The banker was trapped
6	4	The banker got trapped
7	1	The producer was hit by the driver
7	2	The producer got hit by the driver
7	3	The producer was hit
7	4	The producer got hit
8	1	The gymnast was employed by the photographer
8	2	The gymnast got employed by the photographer
8	3	The gymnast was employed
8	4	The gymnast got employed
9	1	The diver was identified by the medic
9	2	The diver got identified by the medic
9	3	The diver was identified
9	4	The diver got identified
10	1	The model was attacked by the guard
10	2	The model got attacked by the guard
10	3	The model was attacked
10	4	The model got attacked
11	1	The painter was paid by the joiner
11	2	The painter got paid by the joiner
11	3	The painter was paid
11	4	The painter got paid
12	1	The miner was freed by the explorer
12	2	The miner got freed by the explorer
12	3	The miner was freed
12	4	The miner got freed
13	1	The scribe was dismissed by the sheriff
13	2	The scribe got dismissed by the sheriff

13	3	The scribe was dismissed
13	4	The scribe got dismissed
14	1	The secretary was punished by the manager
14	2	The secretary got punished by the manager
14	3	The secretary was punished
14	4	The secretary got punished
15	1	The guest was caught by the host
15	2	The guest got caught by the host
15	3	The guest was caught
15	4	The guest got caught
16	1	The teacher was killed by the beekeeper
16	2	The teacher got killed by the beekeeper
16	3	The teacher was killed
16	4	The teacher got killed

Table A2 List of stimuli used in Experiment 2.

Item	Condition	Text
1	1	The designer was shoved by the attacker
1	2	The designer got shoved by the attacker
1	3	The designer was shoved by the edge of the stage
1	4	The designer got shoved by the edge of the stage
1	5	The designer was shoved earlier that morning
1	6	The designer got shoved earlier that morning
2	1	The hairdresser was hurt by the tailor
2	2	The hairdresser got hurt by the tailor
2	3	The hairdresser was hurt by the roadside
2	4	The hairdresser got hurt by the roadside
2	5	The hairdresser was hurt during his day off
2	6	The hairdresser got hurt during his day off
3	1	The minister was elected by the mayor
3	2	The minister got elected by the mayor
3	3	The minister was elected by the end of July
3	4	The minister got elected by the end of July
3	5	The minister was elected after the incident
3	6	The minister got elected after the incident
4	1	The squatter was evicted by the inspector
4	2	The squatter got evicted by the inspector
4	3	The squatter was evicted by late afternoon
4	4	The squatter got evicted by late afternoon
4	5	The squatter was evicted after only an hour
4	6	The squatter got evicted after only an hour
5	1	The composer was seduced by the dancer
5	2	The composer got seduced by the dancer
5	3	The composer was seduced by the end of the session
5	4	The composer got seduced by the end of the session
5	5	The composer was seduced in a dark back lane
5	6	The composer got seduced in a dark back lane
6	1	The fraudster was trapped by the alchemist
6	2	The fraudster got trapped by the alchemist
6	3	The fraudster was trapped by that evening
6	4	The fraudster got trapped by that evening
6	5	The fraudster was trapped for what seemed like hours
6	6	The fraudster got trapped for what seemed like hours
7	1	The jogger was hit by the driver
7	2	The jogger got hit by the driver
7	3	The jogger was hit by the edge of the car park
7	4	The jogger got hit by the edge of the car park
7	5	The jogger was hit after looking suspicious
7	6	The jogger got hit after looking suspicious
8	1	The gymnast was promoted by the instructor
8	2	The gymnast got promoted by the instructor
8	3	The gymnast was promoted by the New Year
8	4	The gymnast got promoted by the New Year
8	5	The gymnast was promoted after a long wait
8	6	The gymnast got promoted after a long wait
9	1	The diver was blamed by the medic
9	2	The diver got blamed by the medic
9	3	The diver was blamed by the end of the day
9	4	The diver got blamed by the end of the day
9	5	The diver was blamed after his past was uncovered
9	6	The diver got blamed after his past was uncovered

10	1	The model was attacked by the guard
10	2	The model got attacked by the guard
10	3	The model was attacked by the riverside
10	4	The model got attacked by the riverside
10	5	The model was attacked inside a nightclub
10	6	The model got attacked inside a nightclub
11	1	The painter was paid by the contractor
11	2	The painter got paid by the contractor
11	3	The painter was paid by the deadline
11	4	The painter got paid by the deadline
11	5	The painter was paid soon after the exhibition
11	6	The painter got paid soon after the exhibition
12	1	The climber was side-tracked by the explorer
12	2	The climber got side-tracked by the explorer
12	3	The climber was side-tracked by the time dawn came
12	4	The climber got side-tracked by the time dawn came
12	5	The climber was side-tracked after several hours
12	6	The climber got side-tracked after several hours
13	1	The scribe was taught by the paladin
13	2	The scribe got taught by the paladin
13	3	The scribe was taught by candle-light
13	4	The scribe got taught by candle-light
13	5	The scribe was taught about many things
13	6	The scribe got taught about many things
14	1	The secretary was punished by the manager
14	2	The secretary got punished by the manager
14	3	The secretary was punished by the end of the week
14	4	The secretary got punished by the end of the week
14	5	The secretary was punished with demotion
14	6	The secretary got punished with demotion
15	1	The guest was caught by the host
15	2	The guest got caught by the host
15	3	The guest was caught by the end of the search
15	4	The guest got caught by the end of the search
15	5	The guest was caught with all the stolen goods
15	6	The guest got caught with all the stolen goods
16	1	The teacher was killed by the madman
16	2	The teacher got killed by the madman
16	3	The teacher was killed by sunset
16	4	The teacher got killed by sunset
16	5	The teacher was killed before the rescue
16	6	The teacher got killed before the rescue
17	1	The ranger was bitten by the wildling
17	2	The ranger got bitten by the wildling
17	3	The ranger was bitten by the edge of the cage
17	4	The ranger got bitten by the edge of the cage
17	5	The ranger was bitten near the edge of the cage
17	6	The ranger got bitten near the edge of the cage
18	1	The player was tackled by an opponent
18	2	The player got tackled by an opponent
18	3	The player was tackled by the end of the game
18	4	The player got tackled by the end of the game
18	5	The player was tackled at the start of the match
18	6	The player got tackled at the start of the match
